# Data on *in vivo* antioxidant, hypolipidemic and hepatoprotective potential of *Thaumatococcus daniellii* (Benn.) Benth leaves

**DOI:** 10.1016/j.dib.2018.08.016

**Published:** 2018-08-10

**Authors:** Shalom Nwodo Chinedu, Franklyn Nonso Iheagwam, Boluwatife Taiwo Makinde, Babajide Oladipo Thorpe, Opeyemi Christianah Emiloju

**Affiliations:** a Department of Biochemistry, College of Science and Technology, Covenant University, P.M.B. 1023, Canaanland, Ota, Ogun State, Nigeria; b Covenant University Public Health and Wellness Research Cluster (CUPHWERC), Covenant University, P.M.B. 1023, Canaanland, Ota, Ogun State, Nigeria

**Keywords:** *Thaumatococcus daniellii*, *in vivo*, Oxidative stress, Antioxidant, Liver injury, Hypolipidemic

## Abstract

This data article reports on the *in vivo* biochemical activity of ethanolic extract of *Thaumatococcus daniellii* (Benn.) Benth leaves (ETD) in male Wistar rats at an oral dose of 500–1500 mg/kg daily for 14 days. Control groups were administered distilled water and Vitamin C (10 mg/kg; b.wt). Indices of oxidative stress, dyslipidemia, liver injury and liver pathology were estimated in the plasma and organs after the investigation period. Oral treatment with ETD increased organ superoxide dismutase (SOD) activity, renal reduced glutathione (GSH) and plasma high density lipoprotein (HDL) concentrations while reducing plasma alanine transaminase (ALT) activity, plasma cholesterol (CHOL), bilirubin (DBIL) and organ malondialdehyde (MDA) concentrations (*P*<0.05). Data was supported by histological report showing no pathologic abnormality. This data indicate ethanolic extract of *T. daniellii* leaves shows antioxidant, hypolipidemic and hepatoprotective potential.

**Specifications Table**

**Table:** 

Subject area	*Biochemistry*
More specific subject area	*Pharmacology, Medicinal and Food Plants*
Type of data	*Table, Text File, Graph, Figure*
How data was acquired	*All data were acquired using a spectrophotometer (Thermo Fisher Scientific, GEN10S, Madison, USA) and weighing balance (Ohaus Corp., PA4202C, New Jersey, USA)*
Data format	*Raw, analyzed and expressed as mean*±*S.D. from five animals.*
Experimental factors	*Ethanolic extract of T. daniellii leaves was prepared and concentrated using a rotary evaporator*
Experimental features	*Lipid profile, antioxidant and liver function parameters in plasma, liver and kidney were examined*
Data source location	*Department of Biochemistry, Covenant University, Ota, Nigeria.*
Data accessibility	*Data is supplied in this article*
Related research article	*S.N. Chinedu, F.N. Iheagwam, C.J. Anichebem, G.B. Ogunnaike, O.C. Emiloju, Antioxidant and biochemical evaluation of Thaumatococcus daniellii seeds in rat, J. Biol. Sci. 17(8) (2017) 381–387.*

**Value of the data**

• The presented data indicate *T. daniellii leaf* possess antioxidant and hypolipidemic properties comparable with vitamin C.
• Data shows *T. daniellii leaf* may not be toxic or injurious to the organs at the tested doses and time frame.
• The data in this article may prove that *T. daniellii leaf* is a natural source of bioactivities with healthy benefits.
• The data is important in promoting an alternative use of *T. daniellii* leaf which is usually discarded as waste.

## 1 Data

**Fig. 1:**
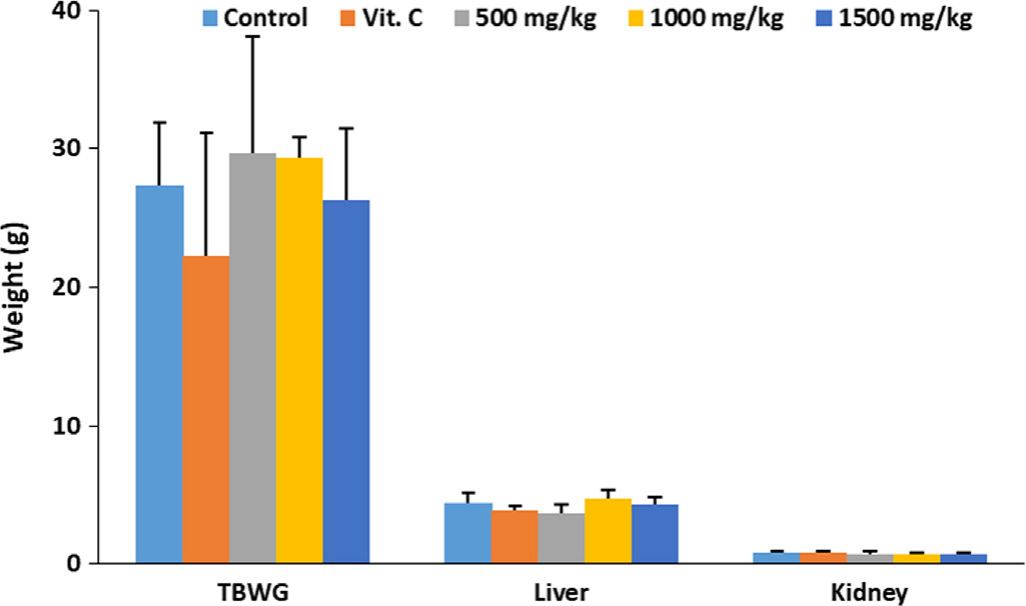
Effect of ETD on animal weight gain and organ weight. Bars are expressed as mean ± S.D. (*n* = 5). TBWG: total body weight gain.

**Fig. 2:**
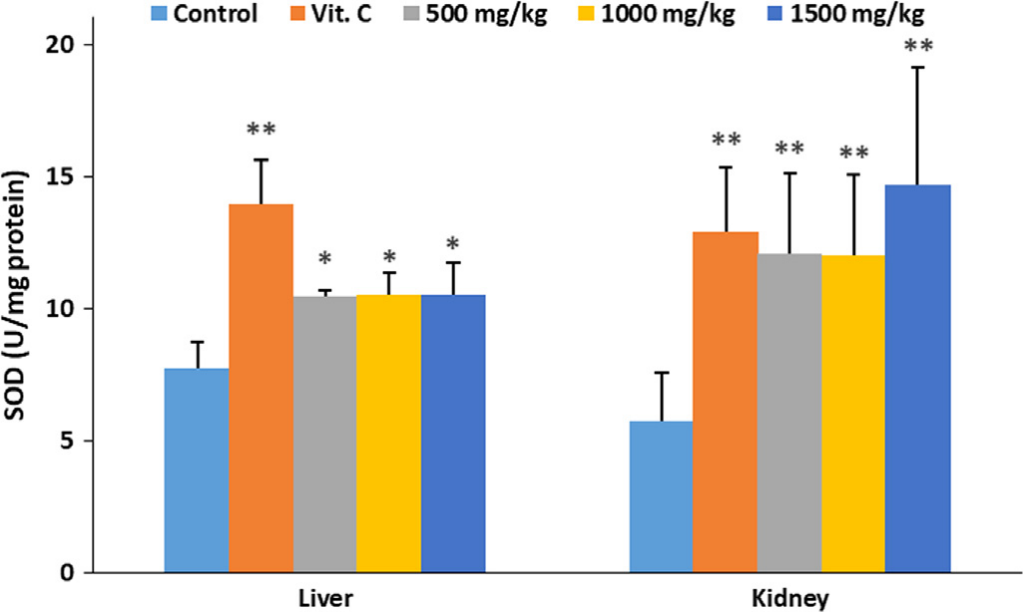
Effect of ETD on liver and kidney SOD concentration. Bars are expressed as mean ± S.D. (*n* = 5). Bars denoted with (*) and (**) are significantly different at *p*< 0.05 and *p*< 0.01 respectively when compared with control group.

**Fig. 3:**
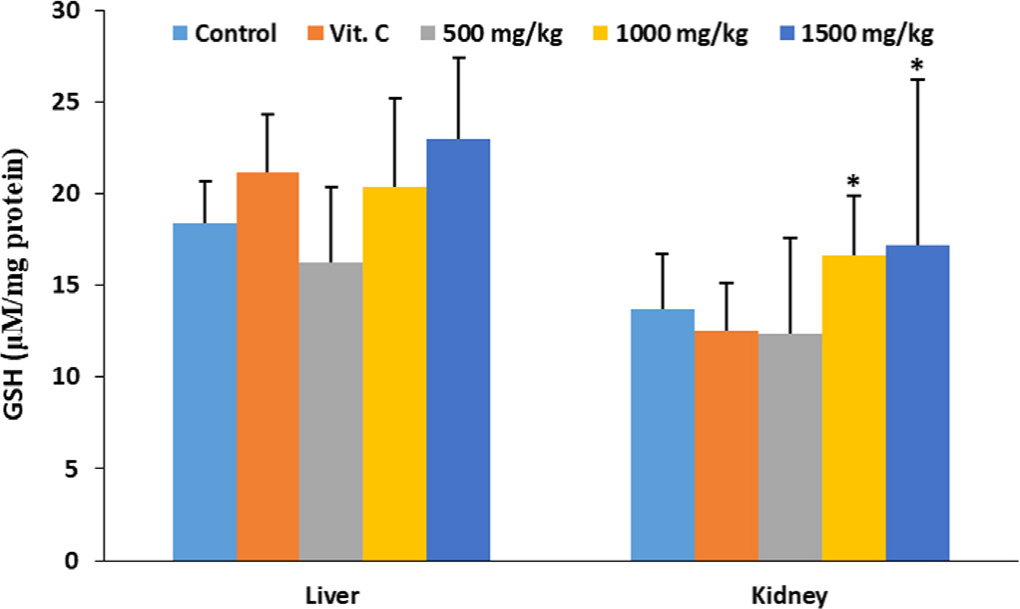
Effect of ETD on liver and kidney GSH concentration. Bars are expressed as mean ± S.D. (*n* = 5). Bars denoted with (*) are significantly different at *p*< 0.05 when compared with the control group.

**Fig. 4:**
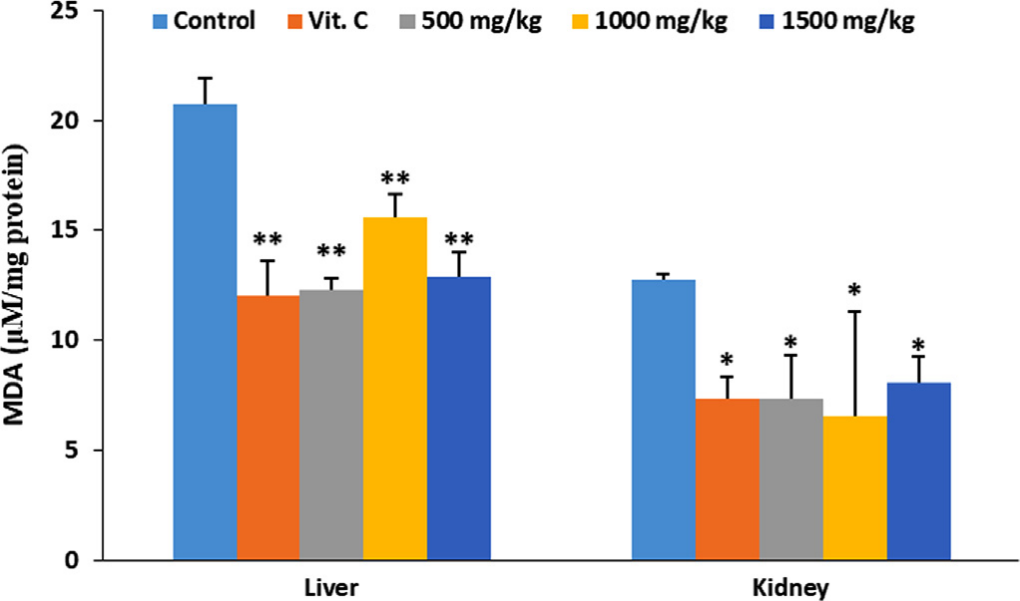
Effect of ETD on liver and kidney MDA concentration. Bars are expressed as mean ± S.D. (*n* = 5). Bars denoted with (*) and (**) are significantly different at *p*< 0.05 and *p*< 0.01 respectively when compared with control group.

**Fig. 5:**
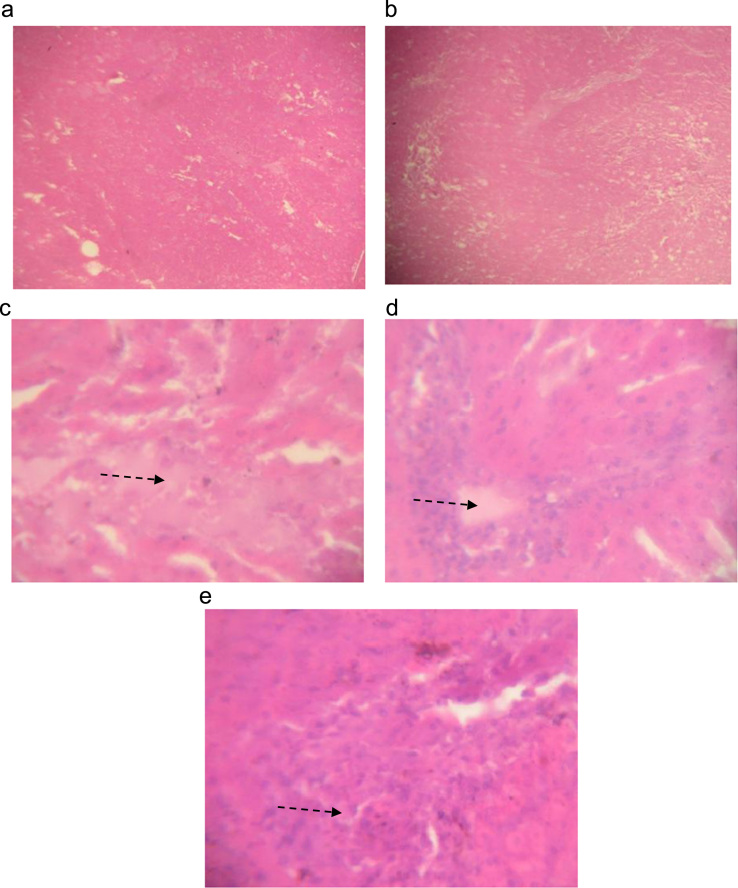
Cross-sectional liver histology of a) Control and b) Vitamin C showing normal histology, while c) ETD (500 mg/kg) d) ETD (1000 mg/kg) e) ETD (1500 mg/kg) shows the sinusoid appear distended with fluid highlighting oedema (dotted arrow) (x400).

The data describes the effect of ethanolic extract of *Thaumatococcus daniellii* leaves (ETD) on oxidative stress parameters, lipid profile, liver function and pathology. Data on the effect of ETD treatment on weight gain, organ weight, superoxide dismutase (SOD) activity, reduced glutathione (GSH) and malondialdehyde (MDA) concentrations are presented in Fig. 1, Fig. 2, Fig. 3, Fig. 4 respectively. Plasma and organ lipid profile and liver function parameters are shown in Table 1, Table 2 respectively. Histological data on changes in rat liver is depicted in Fig. 5.

**Table 1:** Effect of ETD on plasma, liver and kidney lipid profile.

**Treatment and Dose**	**Plasma**	**Liver**	**Kidney**
**Cholesterol (mmol/L)**			
Control	1.33±0.25	0.70±0.42	0.15±0.02
Vitamin C (10 mg/kg)	1.25±0.36	0.59±0.02	0.20±0.11
ETD (500 mg/kg)	1.38±0.25	0.64±0.27	0.14±0.07
ETD (1000 mg/kg)	1.35±0.29	0.67±0.18	0.13±0.02
ETD (1500 mg/kg)	1.18±0.49^*^	0.63±0.31	0.17±0.09
**High-density lipoprotein (mmol/L)**			
Control	1.12±0.27	0.12±0.07	0.05±0.04
Vitamin C (10 mg/kg)	1.69±0.16^*^	0.16±0.02	0.09±0.09
ETD (500 mg/kg)	1.62±0.20^*^	0.22±0.09	0.04±0.02
ETD (1000 mg/kg)	1.64±0.22^*^	0.18±0.02	0.05±0.02
ETD (1500 mg/kg)	1.73±0.11^*^	0.15±0.07	0.07±0.04
**Triglyceride (mmol/L)**			
Control	2.32±0.20	1.27±0.20	0.25±0.31
Vitamin C (10 mg/kg)	2.50±0.36	1.24±0.56	0.25±0.27
ETD (500 mg/kg)	2.36±0.25	1.18±0.45	0.22±0.11
ETD (1000 mg/kg)	2.28±0.38	1.24±0.25	0.19±0.16
ETD (1500 mg/kg)	2.66±0.27	1.13±0.27	0.16±0.07
**Low-density lipoprotein (mmol/L)**			
Control	0.62±0.40	0.54±0.51	0.11±0.11
Vitamin C (10 mg/kg)	0.43±0.34	0.46±0.45	0.08±0.07
ETD (500 mg/kg)	0.53±0.25	0.56±0.27	0.09±0.04
ETD (1000 mg/kg)	0.47±0.18	0.49±0.34	0.11±0.11
ETD (1500 mg/kg)	0.48±0.11	0.53±0.42	0.13±0.11

Data are expressed as mean ± S.D. (*n* = 5). Values denoted with (*) down a column are significantly different at *p*< 0.05 when compared with control group

**Table 2:** Effect of ETD on liver function markers in plasma and liver.

**Treatment and Dose**	**Plasma**	**Liver**
**Alanine transaminase (U/I)**		
Control	17.55±1.92	29.31±9.84
Vitamin C (10 mg/kg)	12.03±6.17^*^	26.55±2.77
ETD (500 mg/kg)	8.78±6.66^*^	26.01±13.84
ETD (1000 mg/kg)	8.61±3.67^*^	31.05±8.36
ETD (1500 mg/kg)	12.32±4.00^*^	28.70±5.32
**Aspartate transaminase (U/I)**		
Control	141.20±62.95	301.30±38.86
Vitamin C (10 mg/kg)	107.94±20.66	315.33±32.80
ETD (500 mg/kg)	137.01±32.36	316.52±23.72
ETD (1000 mg/kg)	107.22±15.56	272.28±121.84
ETD (1500 mg/kg)	125.54±25.96	229.17±96.37
**Total bilirubin (µmol/L)**		
Control	23.31±15.34	28.68±15.21
Vitamin C (10 mg/kg)	20.97±3.44	29.89±3.18
ETD (500 mg/kg)	12.49±7.78^**^	33.49±11.07
ETD (1000 mg/kg)	15.48±5.63^*^	29.13±6.08
ETD (1500 mg/kg)	10.08±4.29^**^	27.51±3.22
**Direct bilirubin (µmol/L)**		
Control	31.52±17.82	50.96±24.13
Vitamin C (10 mg/kg)	36.93±14.71	40.63±9.12
ETD (500 mg/kg)	33.66±18.96	41.64±12.52
ETD (1000 mg/kg)	32.63±24.33	44.24±19.88
ETD (1500 mg/kg)	37.50±9.91	47.95±13.98

Data are expressed as mean ± S.D. (*n* = 5). Values denoted with (*) and (**) down a column are significantly different at *p*< 0.05 and *p*< 0.01 respectively when compared with control group

## 2 Experimental design, materials and methods

### 2.1 Collection of plant samples and extracts preparations

Mature *T. daniellii* leaves were bought from Oja-Ota, Ogun State, Nigeria and identified by Dr J.O. Popoola from Covenant University, Ota, Nigeria. Leaf extract was prepared as reported by Chinedu et al. [1] and Iheagwam et al. [2].

### 2.2 Experimental animals

Twenty-five apparently healthy male Wistar rats aged 4–6 weeks old and weighing between 100 g and 150 g were obtained from Federal University of Agriculture, Abeokuta (FUNAAB). Experimental animals were maintained under standard laboratory conditions and allowed access to rodent pellets (Grace Feeds) and water *ad libitum*. Prior the experiments, they were fasted overnight with access to water. Covenant University Ethics committee Guide for Care and Use of Laboratory Animals was strictly adhered to.

### 2.3 Animal groupings and treatment

The rats were weighed and distributed into 5 groups of 5 animals each. Three of the groups served as the treatment groups and were administered with 500–1500 mg/kg b.wt of ethanolic extract of *Thaumatococcus daniellii* leaves (ETD) for 14 days while the remaining 2 groups which served as the controls were administered 1 mL/kg b.wt of vehicle and 10 mg/kg b.wt of vitamin C (Table 3). After 14 days of oral dosing, the animals were fasted overnight for over 12 h, weighed and sacrificed under mild euthanasia with diethyl ether. Fresh blood was collected by cardiac puncture. Tissue homogenates were prepared as described by Chinedu et al. [1].

**Table 3:** Animal grouping and treatment.

**Group**	**Treatment**	**Dosage**	**(*n* = *x*)**
1	Distilled water	1 mL/kg b.wt	5
2	Vitamin C	10 mg/kg b.wt	5
3	ETD	500 mg/kg b.wt	5
4	ETD	1000 mg/kg b.wt	5
5	ETD	1500 mg/kg b.wt	5

### 2.4 Animal and organ weight

Animal and organ weights were measured as a sign of animal and organ toxicity using weighing balance (Ohaus Corp., PA4202C, New Jersey, USA). Data is depicted in Fig. 1.

### 2.5 Indices of oxidative stress

SOD was determined according to the method of Marklund and Marklund [3]. Reduced glutathione (GSH) was assayed according to the method described by Sedlak and Lindsay [4]. Malondialdehyde (MDA) was evaluated using the method of Buege and Aust [5] as an index of lipid peroxidation. Data is presented in Fig. 2, Fig. 3, Fig. 4 respectively.

### 2.6 Lipid profile

Cholesterol, triglyceride, high- and low-density lipoprotein were determined spectrophotometrically using commercial kits (Randox Lab., England, UK) according to manufacturer׳s instructions. The data outcome is recorded in Table 1.

### 2.7 Liver function parameters

Alanine transaminase, aspartate transaminase, total and direct bilirubin were determined spectrophotometrically using commercial kits according to manufacturer׳s instructions (Randox Lab., England, UK). Raw data are shown in Table 2.

### 2.8 Histopathology

Histopathology of liver tissues were evaluated as reported by Chinedu et al. [1]. The observations are shown in Fig. 5.

### 2.9 Statistical analysis

All data were expressed as mean ± Standard deviation (S.D.). Statistical analysis was carried out using one-way analysis of variance (ANOVA) and Duncan Multiple Range Test (DMRT) considered statistically significant at *p*<0.05 and *p*<0.01.
